# Supplementary material for: Modeling spatiotemporal dynamics of Amblyomma americanum questing activity in the central Great Plains
Source: PLoS One. 2024 Oct 28;19(10):e0304427. doi: 10.1371/journal.pone.0304427 (PMC11515986; doi:10.1371/journal.pone.0304427)
Supplement: S1 Table — Names of variables are as follows: maximum air temperature (tmax), minimum air temperature (tmin), precipitation (prcp), shortwave radiation (srad), water vapor pressure (wvap), and day length (dayl). (DOCX) [file pone.0304427.s005.docx]

**S1 Table. Summary of Pearson product-moment correlations among environmental variables.** Names of variables are as follows: maximum air temperature (tmax), minimum air temperature (tmin), precipitation (prcp), shortwave radiation (srad), water vapor pressure (wvap), and day length (dayl).

|  | dayl | prcp | srad | tmax | tmin | vp |
| --- | --- | --- | --- | --- | --- | --- |
| dayl | – | 0.28 | 0.75 | 0.73 | 0.82 | 0.80 |
| prcp | 0.28 | – | -0.13 | 0.00 | 0.15 | 0.12 |
| srad | 0.75 | -0.13 | – | 0.73 | 0.64 | 0.58 |
| tmax | 0.73 | 0.00 | 0.73 | – | 0.95 | 0.91 |
| tmin | 0.82 | 0.15 | 0.64 | 0.95 | – | 0.97 |
| wvap | 0.80 | 0.12 | 0.58 | 0.91 | 0.97 | – |
