# Supplementary material for: Modeling spatiotemporal dynamics of Amblyomma americanum questing activity in the central Great Plains
Source: PLoS One. 2024 Oct 28;19(10):e0304427. doi: 10.1371/journal.pone.0304427 (PMC11515986; doi:10.1371/journal.pone.0304427)
Supplement: S2 Table — (DOCX) [file pone.0304427.s006.docx]

**S2 Table. Summary of variance summarized by each of the principal components (PCs).**

|  | PC1 | PC2 | PC3 | PC4 | PC5 | PC6 |
| --- | --- | --- | --- | --- | --- | --- |
| Standard deviation | 2.004 | 1.076 | 0.762 | 0.402 | 0.264 | 0.120 |
| Proportion of variance | 0.670 | 0.193 | 0.097 | 0.027 | 0.012 | 0.002 |
| Cumulative proportion | 0.670 | 0.862 | 0.959 | 0.986 | 0.998 | 1.000 |
