# Supplementary material for: Modeling spatiotemporal dynamics of Amblyomma americanum questing activity in the central Great Plains
Source: PLoS One. 2024 Oct 28;19(10):e0304427. doi: 10.1371/journal.pone.0304427 (PMC11515986; doi:10.1371/journal.pone.0304427)
Supplement: S3 Table — Names of variables are as follows: maximum air temperature (tmax), minimum air temperature (tmin), precipitation (prcp), shortwave radiation (srad), water vapor pressure (wvap), and day length (dayl). (DOCX) [file pone.0304427.s007.docx]

**S3 Table. Summary of loadings of raw climatic variables on each of the 6 principal components (PCs).** Names of variables are as follows: maximum air temperature (tmax), minimum air temperature (tmin), precipitation (prcp), shortwave radiation (srad), water vapor pressure (wvap), and day length (dayl).

|  | PC1 | PC2 | PC3 | PC4 | PC5 | PC6 |
| --- | --- | --- | --- | --- | --- | --- |
| dayl | 0.46 | -0.03 | 0.38 | 0.63 | 0.45 | -0.21 |
| prcp | 0.12 | 0.84 | 0.46 | -0.23 | -0.14 | -0.01 |
| srad | 0.36 | -0.49 | 0.55 | -0.19 | -0.50 | 0.18 |
| tmax | 0.47 | -0.10 | -0.19 | -0.60 | 0.26 | -0.56 |
| tmin | 0.48 | 0.10 | -0.26 | -0.14 | 0.29 | 0.76 |
| vp | 0.44 | 0.20 | -0.48 | 0.36 | -0.61 | -0.17 |
