# Supplementary material for: Modeling spatiotemporal dynamics of Amblyomma americanum questing activity in the central Great Plains
Source: PLoS One. 2024 Oct 28;19(10):e0304427. doi: 10.1371/journal.pone.0304427 (PMC11515986; doi:10.1371/journal.pone.0304427)
Supplement: S4 Table — “Higher” and “lower” indicate that the observed value of the occurrences of the tick with regard to the environmental dimension in question fell in the upper or lower 2.5% of the null distribution, respectively. (DOCX) [file pone.0304427.s008.docx]

**S4 Table. Summary of univariate tests for the existence of a distinguishable environmental bias (ecological niche) of the tick species *Amblyomma americanum*, for each life stage of the tick species separately, relative to all of the sampling events in this study.** “Higher” and “lower” indicate that the observed value of the occurrences of the tick with regard to the environmental dimension in question fell in the upper or lower 2.5% of the null distribution, respectively.

|  |  | Niche position | | Niche breadth | |
| --- | --- | --- | --- | --- | --- |
| Life stage | Variable | Mean | Median | Standard deviation | Range |
| Nymph | Day length | higher | higher | lower | lower |
|  | Precipitation | higher | higher | – | higher |
|  | Solar radiation | higher | higher | lower | – |
|  | Maximum temperature | higher | higher | lower | – |
|  | Minimum temperature | higher | higher | lower | lower |
|  | Vapor pressure | higher | higher | lower | – |
| Adult | Day length | higher | higher | lower | lower |
|  | Precipitation | higher | higher | – | – |
|  | Solar radiation | higher | higher | lower | – |
|  | Maximum temperature | higher | – | lower | – |
|  | Minimum temperature | higher | higher | – | – |
|  | Vapor pressure | higher | higher | – | – |
| Larva | Day length | – | – | lower | lower |
|  | Precipitation | lower | – | – | – |
|  | Solar radiation | higher | – | lower | lower |
|  | Maximum temperature | higher | higher | lower | – |
|  | Minimum temperature | higher | higher | lower | – |
|  | Vapor pressure | higher | higher | – | – |
