# Supplementary material for: Modeling spatiotemporal dynamics of Amblyomma americanum questing activity in the central Great Plains
Source: PLoS One. 2024 Oct 28;19(10):e0304427. doi: 10.1371/journal.pone.0304427 (PMC11515986; doi:10.1371/journal.pone.0304427)
Supplement: S5 Table — Predictor variables are shown; “^2” indicates quadratic terms in a model. Values for AUC, sensitivity, specificity, and TSS are averages deriving from the 10-kfold evluation process. Threshold indicates the value used to test model sensitivity and specificity. AUC = area under the receiver operative characteristic curve; TSS = true skill statistic; AIC = Akaike information criterion; Δ AIC = delta AIC; AIC γ = AIC weight. (DOCX) [file pone.0304427.s009.docx]

**S5 Table. “Best” models selected in the process of model calibration and evaluation performed per life stage.** Predictor variables are shown; “^2” indicates quadratic terms in a model. Values for AUC, sensitivity, specificity, and TSS are averages deriving from the 10-kfold evluation process. Threshold indicates the value used to test model sensitivity and specificity. AUC = area under the receiver operative characteristic curve; TSS = true skill statistic; AIC = Akaike information criterion; Δ AIC = delta AIC; AIC γ = AIC weight.

| Stage | ID | Predictors | Threshold | AUC | Sensitivity | Specificity | TSS | AIC | Δ AIC | AIC γ |
| --- | --- | --- | --- | --- | --- | --- | --- | --- | --- | --- |
| Nymph | 1 | PC1, PC2, PC4, PC5, I(PC1^2), I(PC6^2) | 0.55 | 0.88 | 0.89 | 0.85 | 0.74 | 140.67 | 1.85 | 0.06 |
|  | 2 | PC1, PC3, PC4, I(PC1^2), I(PC3^2), I(PC6^2) | 0.50 | 0.88 | 0.91 | 0.83 | 0.74 | 139.71 | 0.89 | 0.10 |
|  | 3 | PC1, PC3, PC4, I(PC1^2), I(PC6^2) | 0.47 | 0.88 | 0.90 | 0.83 | 0.73 | 139.34 | 0.52 | 0.12 |
|  | 4 | PC1, PC3, PC4, PC5, I(PC1^2), I(PC3^2), I(PC6^2) | 0.54 | 0.88 | 0.88 | 0.85 | 0.73 | 140.60 | 1.78 | 0.06 |
|  | 5 | PC1, PC3, PC4, PC5, I(PC1^2), I(PC6^2) | 0.53 | 0.89 | 0.89 | 0.84 | 0.74 | 139.99 | 1.18 | 0.09 |
|  | 6 | PC1, PC4, I(PC1^2), I(PC3^2), I(PC6^2) | 0.48 | 0.87 | 0.89 | 0.85 | 0.74 | 140.13 | 1.32 | 0.08 |
|  | 7 | PC1, PC4, I(PC1^2), I(PC6^2) | 0.54 | 0.88 | 0.87 | 0.86 | 0.74 | 139.48 | 0.67 | 0.11 |
|  | 8 | PC1, PC4, PC5, I(PC1^2), I(PC3^2), I(PC6^2) | 0.52 | 0.88 | 0.89 | 0.83 | 0.72 | 139.73 | 0.91 | 0.10 |
|  | 9 | PC1, PC4, PC5, I(PC1^2), I(PC5^2), I(PC6^2) | 0.57 | 0.88 | 0.89 | 0.85 | 0.74 | 140.72 | 1.90 | 0.06 |
|  | 10 | PC1, PC4, PC5, I(PC1^2), I(PC6^2) | 0.52 | 0.89 | 0.90 | 0.83 | 0.73 | 138.82 | 0.00 | 0.16 |
|  | 11 | PC1, PC4, PC5, PC6, I(PC1^2), I(PC6^2) | 0.53 | 0.88 | 0.90 | 0.83 | 0.73 | 140.62 | 1.81 | 0.06 |
| Adult | 1 | PC1, PC2, PC3, PC4, PC5 | 0.51 | 0.88 | 0.85 | 0.88 | 0.72 | 157.53 | 0.00 | 0.15 |
|  | 2 | PC1, PC2, PC3, PC4, PC5, I(PC1^2) | 0.48 | 0.88 | 0.86 | 0.86 | 0.72 | 159.53 | 2.00 | 0.06 |
|  | 3 | PC1, PC2, PC3, PC4, PC5, I(PC2^2) | 0.50 | 0.88 | 0.84 | 0.90 | 0.74 | 159.20 | 1.67 | 0.07 |
|  | 4 | PC1, PC2, PC3, PC4, PC5, I(PC2^2), I(PC4^2) | 0.51 | 0.88 | 0.86 | 0.91 | 0.77 | 159.27 | 1.74 | 0.06 |
|  | 5 | PC1, PC2, PC3, PC4, PC5, I(PC4^2) | 0.47 | 0.89 | 0.89 | 0.86 | 0.75 | 157.60 | 0.07 | 0.15 |
|  | 6 | PC1, PC2, PC3, PC4, PC5, PC6 | 0.56 | 0.88 | 0.81 | 0.91 | 0.72 | 159.39 | 1.86 | 0.06 |
|  | 7 | PC1, PC2, PC3, PC4, PC5, PC6, I(PC4^2) | 0.49 | 0.88 | 0.85 | 0.90 | 0.75 | 159.20 | 1.67 | 0.07 |
|  | 8 | PC1, PC3, PC4, PC5, I(PC2^2) | 0.51 | 0.88 | 0.84 | 0.91 | 0.75 | 157.96 | 0.43 | 0.12 |
|  | 9 | PC1, PC3, PC4, PC5, I(PC2^2), I(PC4^2) | 0.54 | 0.88 | 0.84 | 0.93 | 0.76 | 157.82 | 0.29 | 0.13 |
|  | 10 | PC1, PC3, PC4, PC5, I(PC4^2) | 0.49 | 0.88 | 0.87 | 0.90 | 0.77 | 158.90 | 1.37 | 0.08 |
|  | 11 | PC1, PC3, PC4, PC5, PC6, I(PC2^2), I(PC4^2) | 0.54 | 0.88 | 0.84 | 0.93 | 0.76 | 159.53 | 2.00 | 0.06 |
| Larva | 1 | PC1, PC2, PC3, PC6, I(PC1^2), I(PC4^2) | 0.19 | 0.83 | 0.87 | 0.86 | 0.73 | 140.15 | 1.04 | 0.15 |
|  | 2 | PC1, PC3, PC4, PC6, I(PC1^2), I(PC4^2) | 0.16 | 0.83 | 0.90 | 0.81 | 0.71 | 141.07 | 1.96 | 0.09 |
|  | 3 | PC1, PC3, PC5, PC6, I(PC1^2), I(PC4^2) | 0.18 | 0.83 | 0.90 | 0.80 | 0.70 | 140.80 | 1.70 | 0.11 |
|  | 4 | PC1, PC3, PC6, I(PC1^2), I(PC2^2), I(PC4^2) | 0.16 | 0.83 | 0.90 | 0.81 | 0.71 | 140.55 | 1.45 | 0.12 |
|  | 5 | PC1, PC3, PC6, I(PC1^2), I(PC3^2), I(PC4^2) | 0.18 | 0.83 | 0.87 | 0.83 | 0.70 | 140.78 | 1.68 | 0.11 |
|  | 6 | PC1, PC3, PC6, I(PC1^2), I(PC4^2) | 0.17 | 0.84 | 0.90 | 0.80 | 0.70 | 139.10 | 0.00 | 0.25 |
|  | 7 | PC1, PC3, PC6, I(PC1^2), I(PC4^2), I(PC5^2) | 0.16 | 0.83 | 0.90 | 0.82 | 0.72 | 141.04 | 1.93 | 0.09 |
|  | 8 | PC1, PC3, PC6, I(PC1^2), I(PC4^2), I(PC6^2) | 0.17 | 0.84 | 0.90 | 0.80 | 0.70 | 141.10 | 2.00 | 0.09 |
