# Supplementary figures and images for: Modeling spatiotemporal dynamics of Amblyomma americanum questing activity in the central Great Plains
Source: PLoS One. 2024 Oct 28;19(10):e0304427. doi: 10.1371/journal.pone.0304427 (PMC11515986; doi:10.1371/journal.pone.0304427)

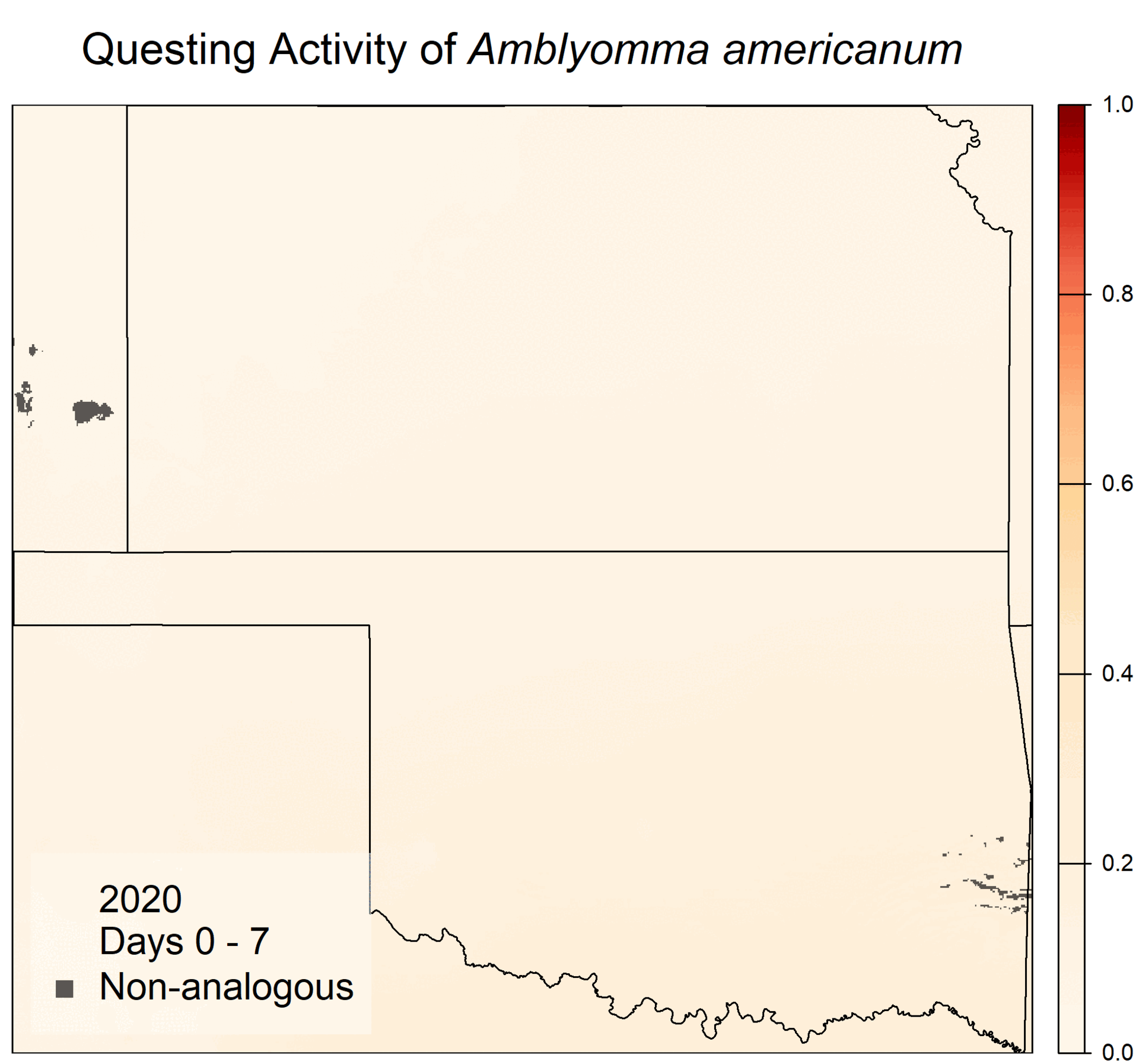

Supplement: S1 File — Time steps are 8-day periods throughout each year. (GIF) [file pone.0304427.s010.gif]

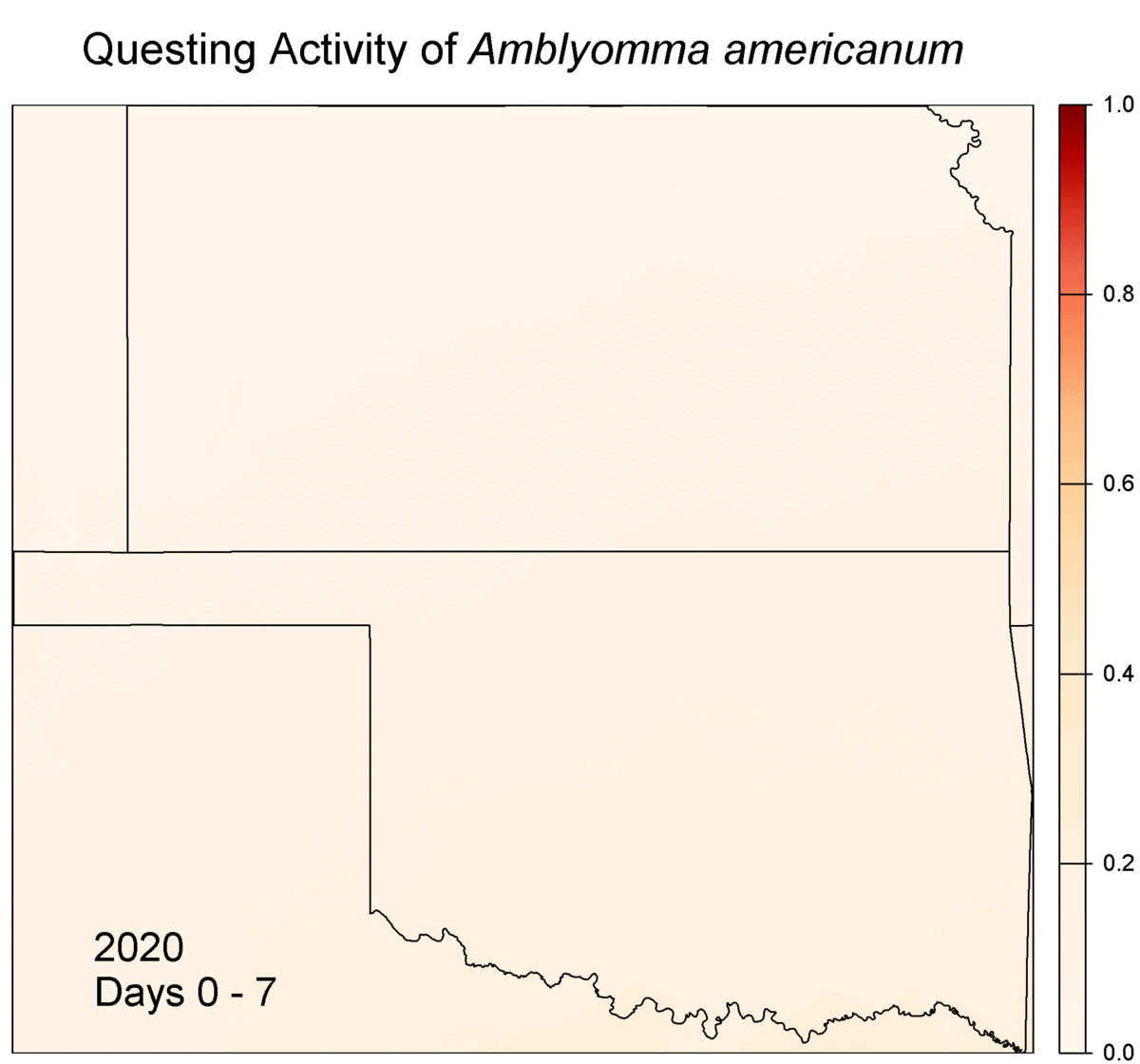

Supplement: S2 File — Time steps are 8-day periods throughout each year. (GIF) [file pone.0304427.s011.gif]

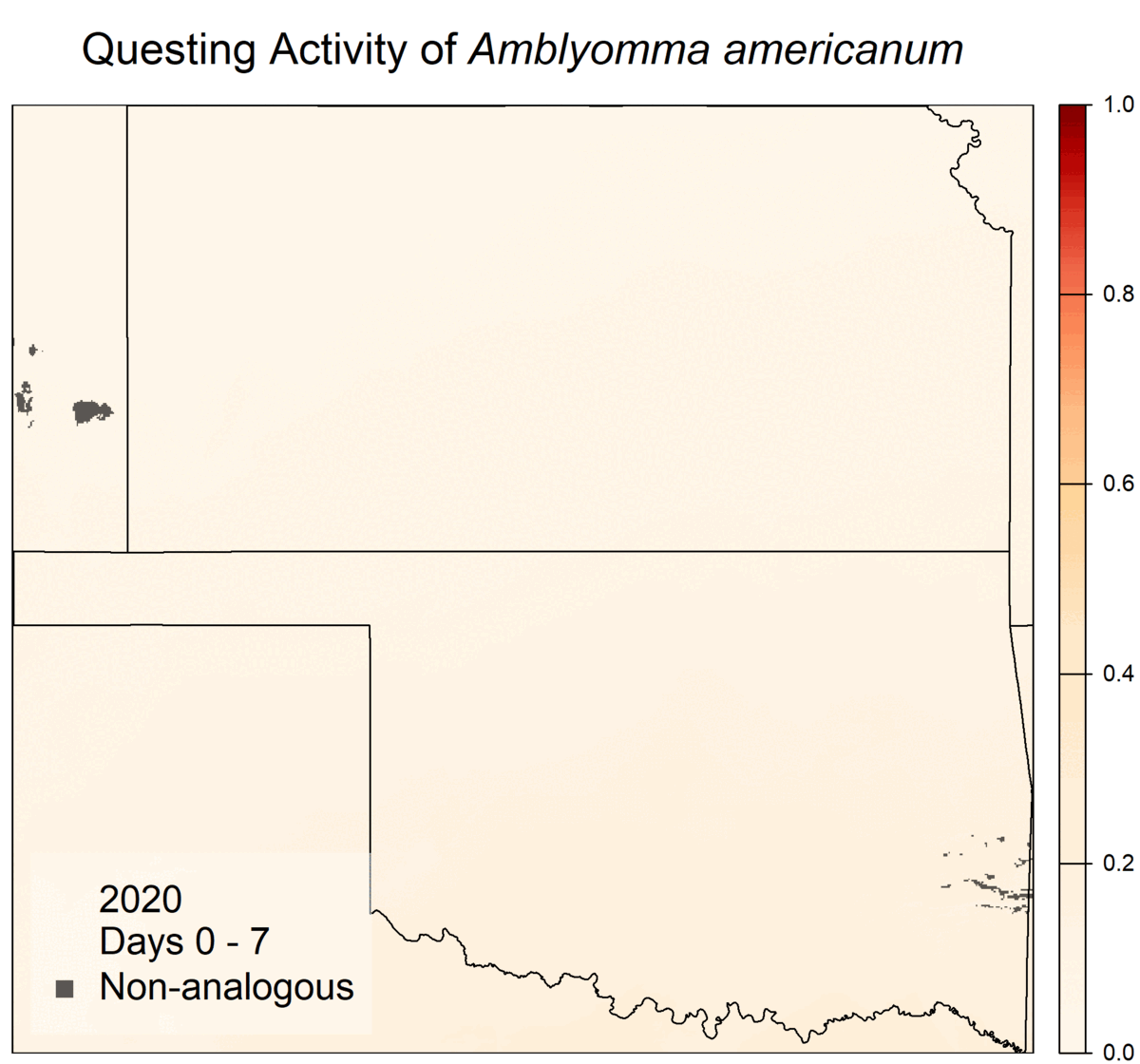

Supplement: S3 File — Time steps are 8-day periods throughout each year. (GIF) [file pone.0304427.s012.gif]

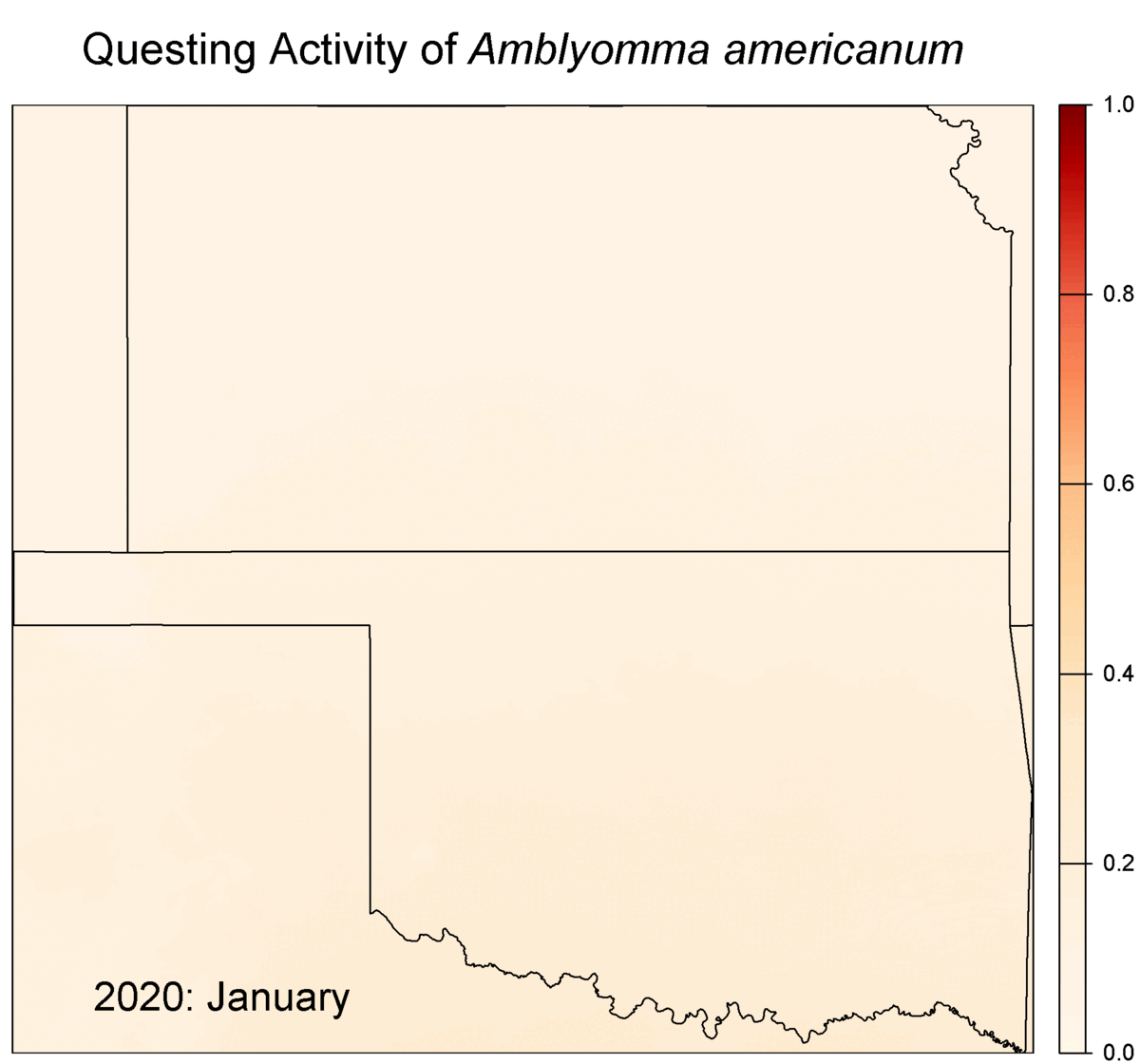

Supplement: S4 File — Time steps are months throughout each year. (GIF) [file pone.0304427.s013.gif]

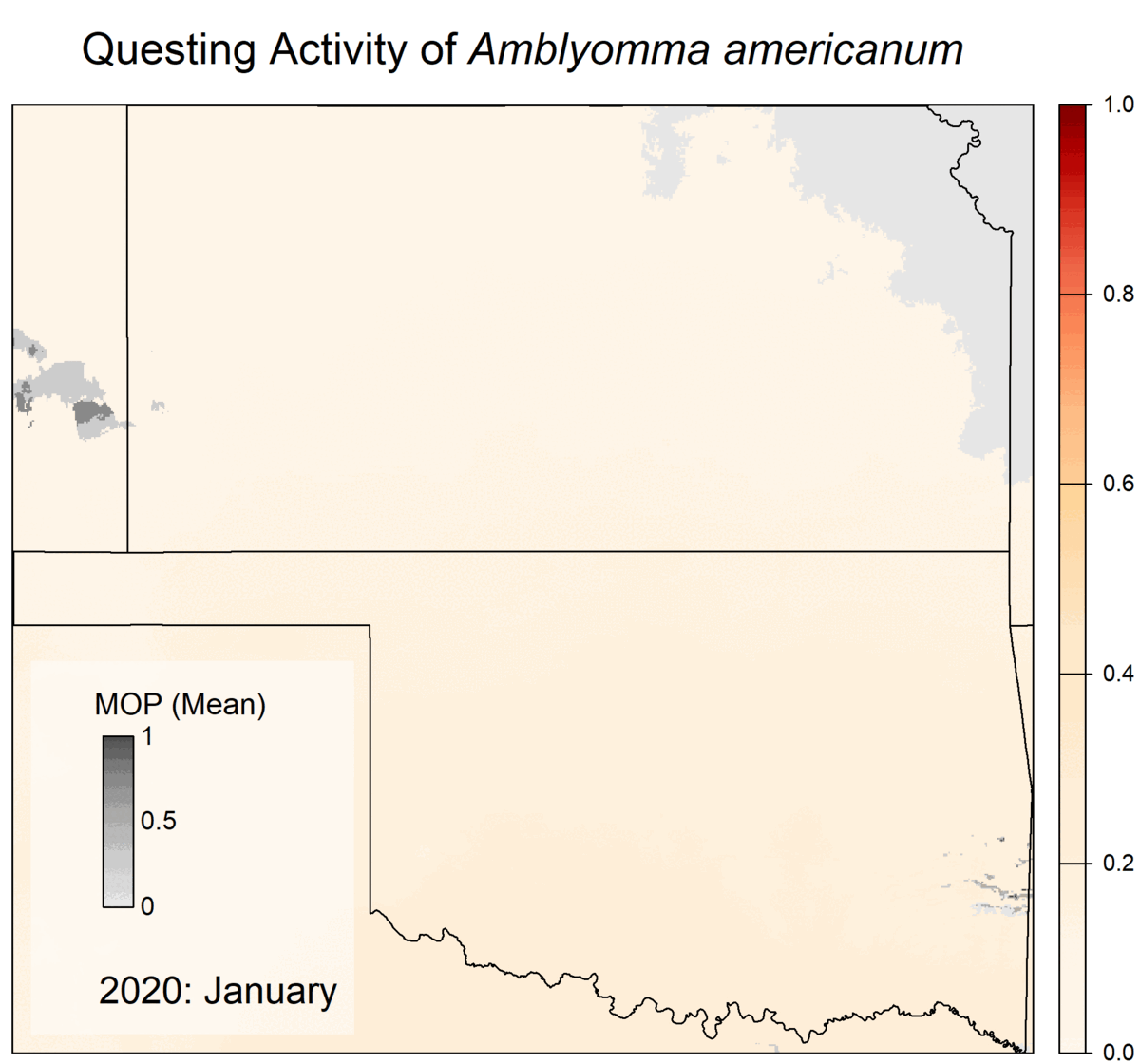

Supplement: S5 File — Time steps are months throughout each year. (GIF) [file pone.0304427.s014.gif]

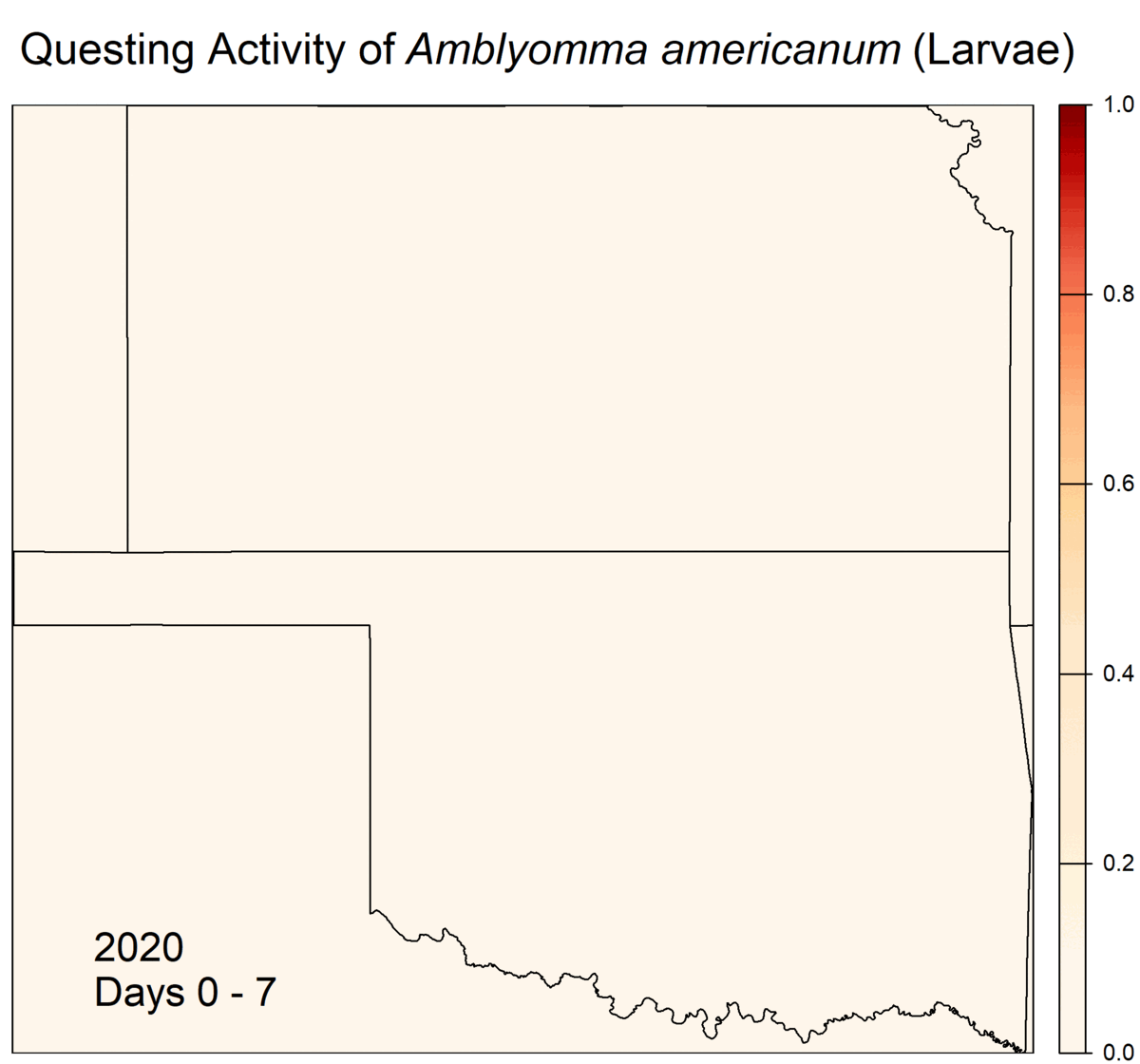

Supplement: S6 File — Time steps are 8-day periods throughout each year. (GIF) [file pone.0304427.s015.gif]

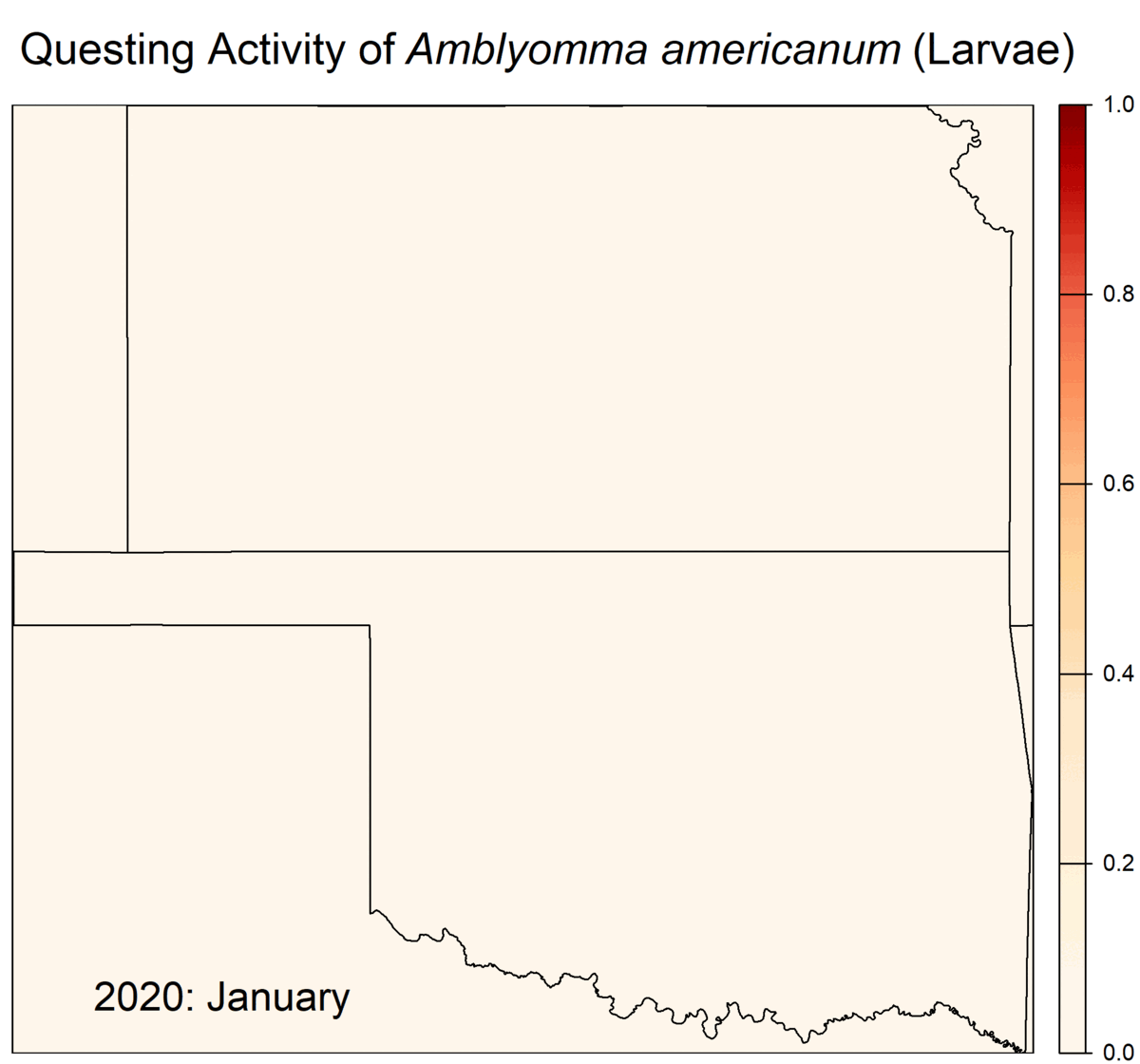

Supplement: S7 File — Time steps are months throughout each year. (GIF) [file pone.0304427.s016.gif]

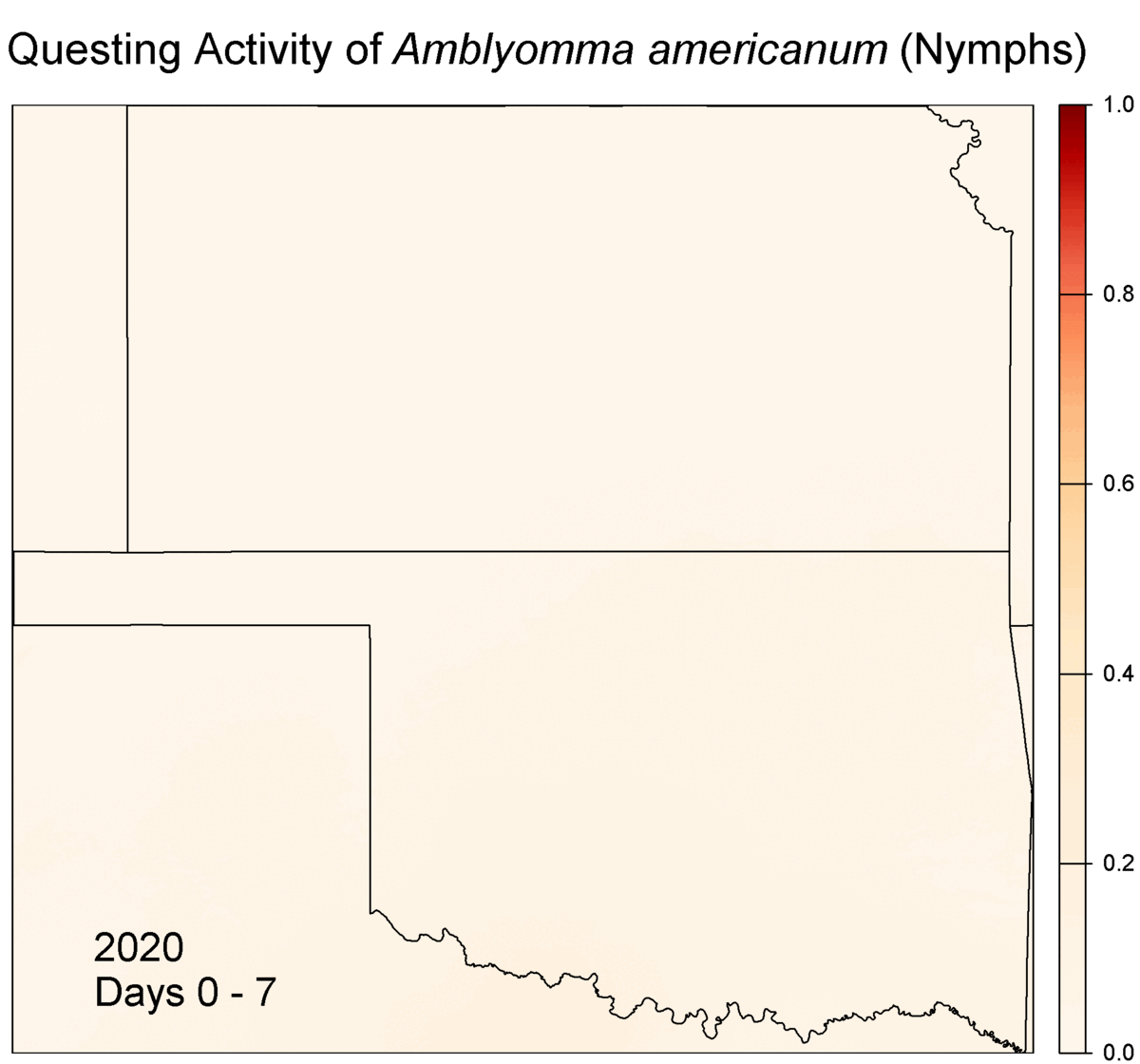

Supplement: S8 File — Time steps are 8-day periods throughout each year. (GIF) [file pone.0304427.s017.gif]

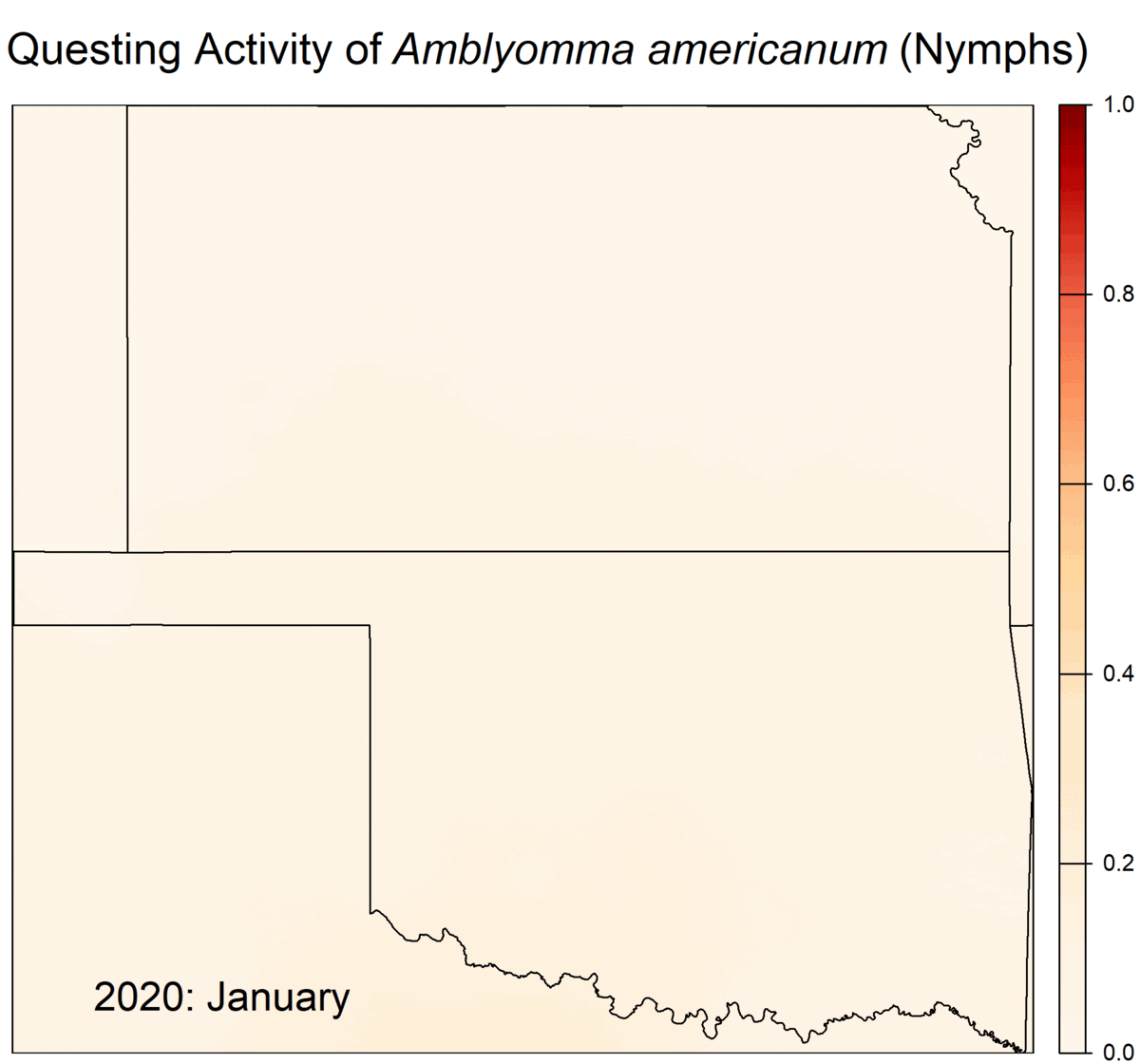

Supplement: S9 File — Time steps are months throughout each year. (GIF) [file pone.0304427.s018.gif]

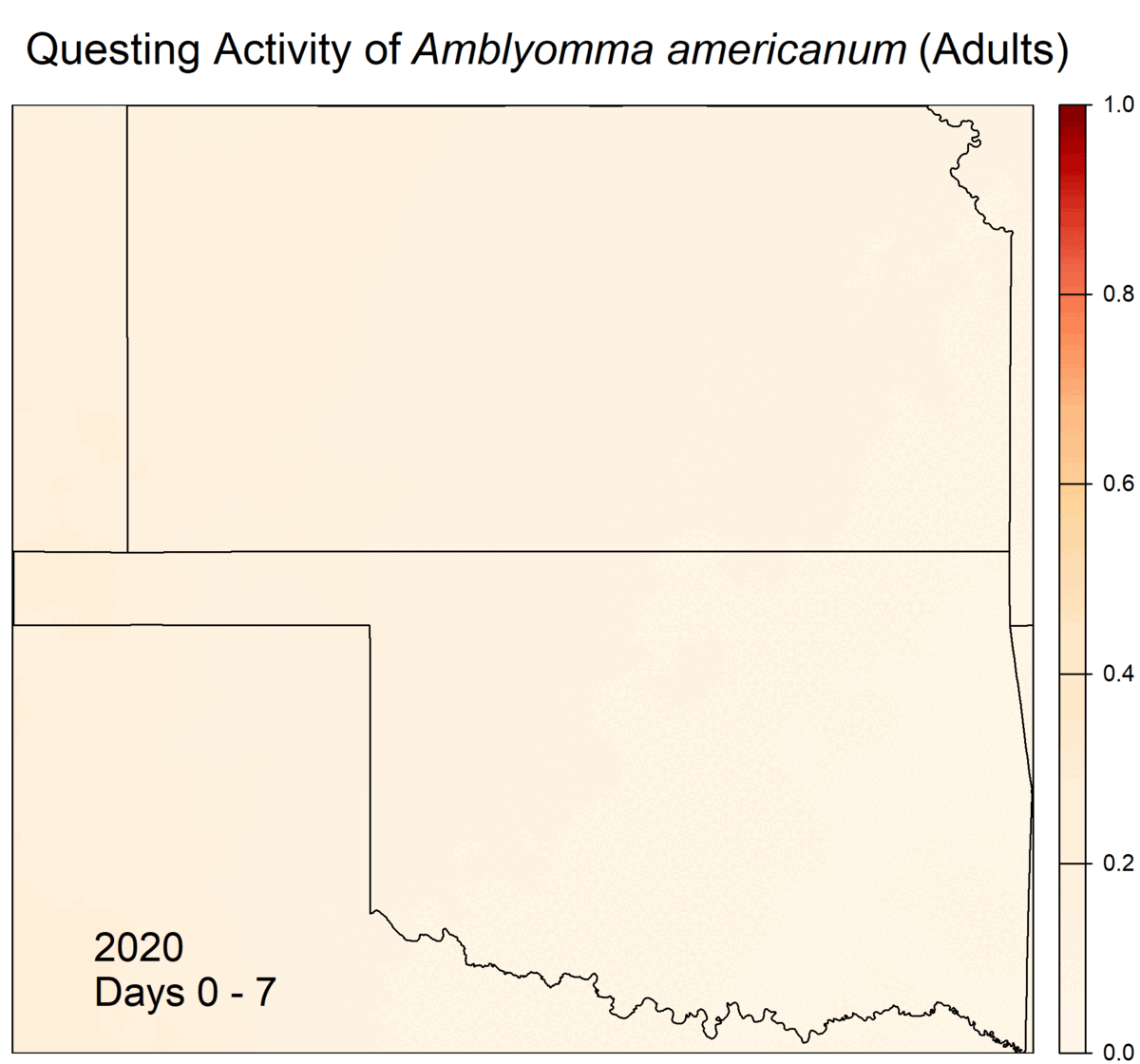

Supplement: S10 File — Time steps are 8-day periods throughout each year. (GIF) [file pone.0304427.s019.gif]

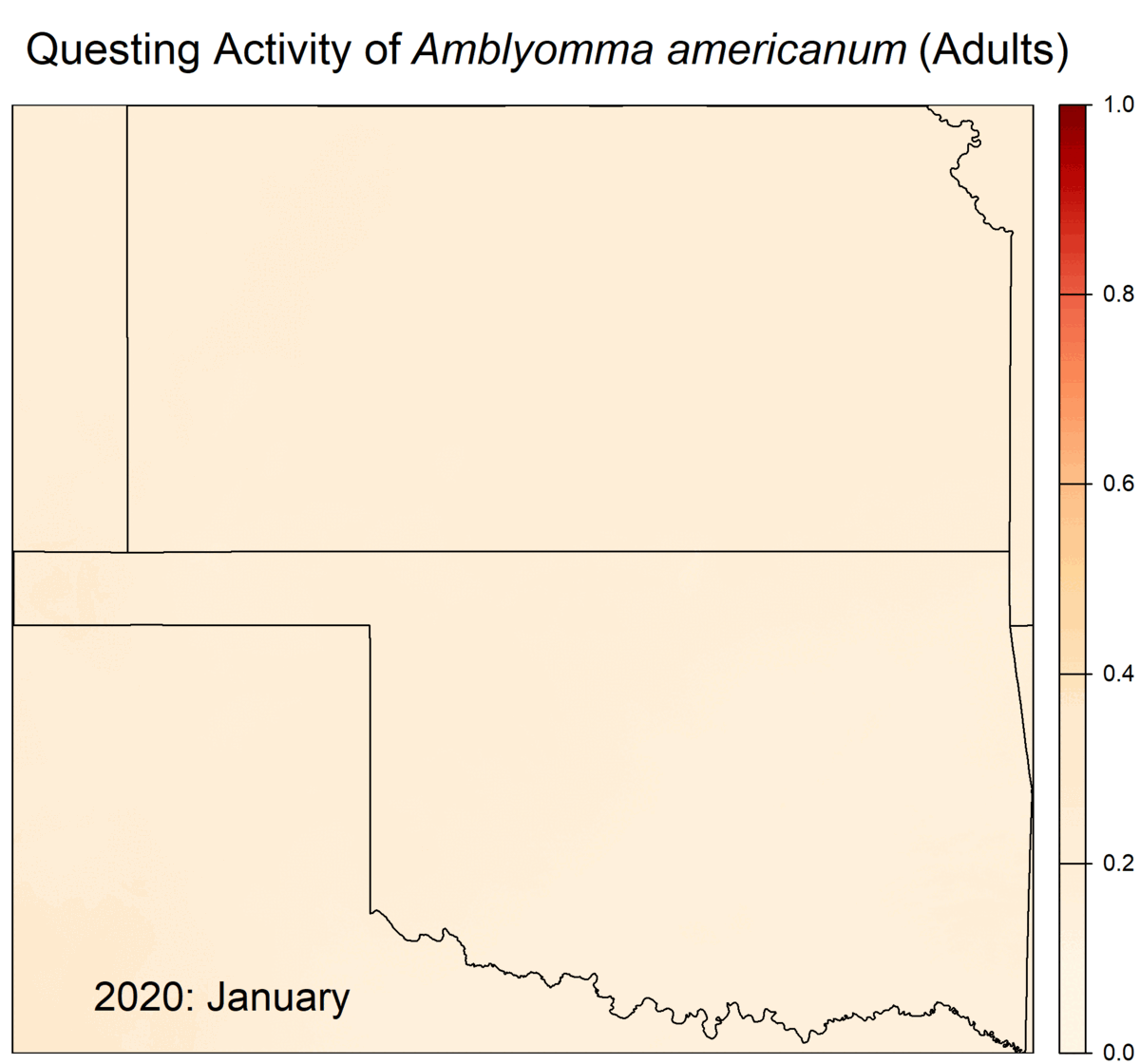

Supplement: S11 File — Time steps are months throughout each year. (GIF) [file pone.0304427.s020.gif]
